# Cellular hierarchy for understanding heterogeneity of acute myeloid leukaemia with t(8;21)/RUNX1‐RUNX1T1

**DOI:** 10.1002/cti2.70042

**Published:** 2025-07-02

**Authors:** Yibo Wu, Xiaolin Yuan, Xiaoyu Lai, Lizhen Liu, Yue Liang, Lihong Ni, Luxin Yang, Shanshan Hu, Jimin Shi, Jian Yu, Yanmin Zhao, Weiyan Zheng, Jie Sun, Yuanyuan Zhu, Wenjun Wu, Zhen Cai, He Huang, Shanshan Pei, Yi Luo

**Affiliations:** ^1^ Bone Marrow Transplantation Center, The First Affiliated Hospital Zhejiang University School of Medicine & Liangzhu Laboratory Hangzhou China; ^2^ Institute of Hematology Zhejiang University Hangzhou China; ^3^ Zhejiang Province Engineering Research Center for Stem Cell and Immunity Therapy Hangzhou China; ^4^ Department of Hematology, Zhejiang Cancer Hospital & Hangzhou Institute of Medicine (HIM) Chinese Academy of Sciences Hangzhou China; ^5^ Department of Hematology, Affiliated with Jinhua Hospital Zhejiang University School of Medicine Jinhua China; ^6^ Department of Hematology Affiliated with Jinhua Hospital, Wenzhou Medical University Jinhua China

**Keywords:** acute myeloid leukaemia, AML, differentiation status, KIT, RUNX1‐RUNX1T1, t(8;21)

## Abstract

**Objectives:**

Differentiation hierarchies in myeloid malignancies influence therapeutic response and prognosis. Acute myeloid leukaemia (AML) with t(8;21) is one of the most recurrent genetic subtypes of AML and is considered a distinct entity with shared characteristics. However, clinical outcomes remain markedly heterogeneous. This study aimed to investigate the relationship between leukaemic arrest at specific differentiation stages, genomic profiles and clinical outcomes in t(8;21) AML.

**Methods:**

We conducted a retrospective study involving 338 patients with t(8;21) AML from three clinical centres in China. Patients received either chemotherapy alone (49.11%, *n* = 166) or chemotherapy followed by allogeneic haematopoietic stem cell transplantation (allo‐HSCT; 41.72%, *n* = 141). Immunophenotypic profiling classified patients into progenitor subgroups: MPP (20.12%, *n* = 68), lymphoid‐primed multi‐potent progenitor (14.50%, *n* = 49), CMP (12.72%, *n* = 43), GMP (24.85%, *n* = 84) and GP/MP (10.36%, *n* = 35). Based on differentiation stage, patients were categorised as primitive (Immuno‐Prim; 47.34%, *n* = 160) or monocytic (Immuno‐Mono; 35.21%, *n* = 119).

**Results:**

The Immuno‐Mono group was associated with lower 2‐year overall survival (OS) and a higher 2‐year cumulative incidence of relapse (CIR) compared to the Immuno‐Prim group. Patients with a KIT mutation had poorer 2‐year OS and higher 2‐year CIR than those without the mutation. In the allo‐HSCT cohort, the Immuno‐Mono group continued to show lower 2‐year OS and higher 2‐year CIR relative to the Immuno‐Prim group. Neither gene mutations (aside from KIT) nor chromosomal losses significantly affected OS or CIR.

**Conclusions:**

Leukaemic differentiation stage independently predicts post‐treatment outcomes in t(8;21) AML. Arrest at specific myeloid stages correlates significantly with genetic aberrations, clinical presentation, therapeutic response and survival.

## Graphical abstract

In this multi‐centre study of 338 t(8;21) acute myeloid leukaemia patients, we demonstrate that leukaemic arrest at monocytic differentiation stages (Immuno‐Mono) predicts significantly worse 2‐year survival and higher relapse rates than primitive‐stage arrest (Immuno‐Prim), independent of therapy. We further identify KIT mutations as compounding adverse factors and show that allogeneic transplantation fails to overcome Immuno‐Mono‐associated risk, establishing differentiation arrest as a key prognostic determinant.

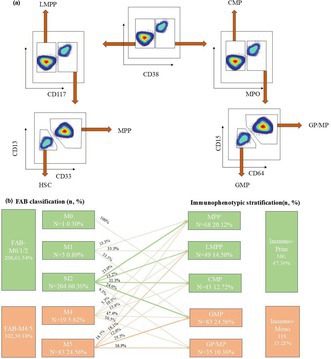

## Introduction

Acute myeloid leukaemia (AML) remains a challenging disease with a poor prognosis.^1^, ^2^, ^3^ Recent advances in single‐cell transcriptomics have revealed the polyclonal and dynamic heterogeneity of AML.^4^, ^5^, ^6^ The differentiation hierarchies of myeloid malignancies can directly influence drug responses and clinical outcomes.^7^, ^8^ Genetic diversity may contribute to phenotypic heterogeneity.^9^ AML with t(8;21) is a chromosomal translocation that encodes an oncoprotein with both mutational and transcriptional functions that drive leukaemogenesis.^10^ It represents one of the most frequently recurring genetic subtypes of AML, accounting for approximately 4–8% of all AML cases. AML with t(8;21)/RUNX1–RUNX1T1 is considered a distinct entity within AML, typically exhibiting shared characteristics. It is commonly associated with the M2 subtype of the French‐American‐British (FAB) classification, frequent expression of CD19, a high prevalence of *KIT* mutations, and additional cytogenetic abnormalities, such as loss of sex chromosomes, as reported in previous studies.^10^, ^11^, ^12^ Despite its classification as a favorable‐risk subtype in the 2022 European LeukemiaNet (ELN) guidelines,^13^ AML with t(8;21) demonstrates considerable clinical heterogeneity. Only 45–70% of patients achieve long‐term disease‐free survival, and approximately 30–40% relapse after attaining complete remission (CR). Some patients even experience relapse following allogeneic haematopoietic stem cell transplantation (allo‐HSCT), often with poor outcomes.^14^ Given the known influence of the developmental stage of leukaemic cells on drug responses and clinical prognosis.^9^, ^15^ AML with t(8;21)/RUNX1–RUNX1T1 provides a compelling model to investigate whether phenotypically defined stages of leukaemic differentiation are associated with specific mutations, treatment responses and clinical outcomes.

In this study, we conducted a comprehensive phenotypic, cytogenetic, molecular and therapeutic response analysis in a large cohort of patients with AML harbouring t(8;21)/RUNX1–RUNX1T1, aiming to elucidate the relationship between leukaemic differentiation stage, genomic features and clinical outcomes.

## Results

### Patients' characteristics

**Figure 1:**
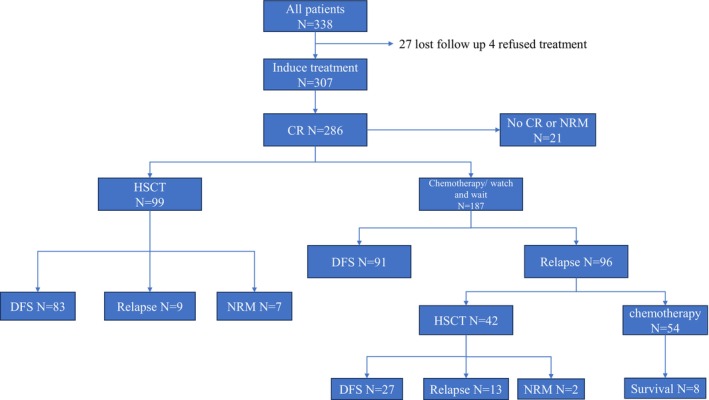
Schedule of acute myeloid leukaemia (AML) patients with t(8;21) accepting treatment.

A total of 338 patients with AML harbouring the t(8;21) translocation were enrolled in this study. The median age at diagnosis was 44 years (range: 5–82), and the median follow‐up duration was 874 days (range: 61–3566). Of the total cohort, 166 patients (49.11%) received chemotherapy alone, while 141 patients (41.72%) underwent allogeneic haematopoietic stem cell transplantation (allo‐HSCT). Additionally, 27 patients (7.99%) were lost to follow‐up, and 4 patients (1.18%) declined treatment following initial diagnosis. During the treatment course, 105 patients (31.07%) experienced disease relapse. At the time of last follow‐up, 91 patients (26.92%) remained disease‐free without having undergone allo‐HSCT, while 110 patients (32.54%) were disease‐free following allo‐HSCT. A total of 22 patients (6.51%) relapsed after transplantation. Among the transplanted patients, 99 (29.29%) underwent allo‐HSCT in first complete remission (CR1), of whom 83 (24.56%) remained disease‐free and 9 (2.66%) relapsed. An additional 42 patients (12.43%) underwent allo‐HSCT in second remission (CR2, post‐relapse), with 27 (7.99%) achieving disease‐free survival and 13 (3.85%) experiencing relapse. The treatment pathways for all patients are illustrated in Figure 1. Regarding cytogenetics, 244 patients (72.19%) exhibited the classical t(8;21)(q22;q22) karyotype, while 32 patients (9.47%) had a normal karyotype and 21 patients (6.21%) had a complex karyotype. Among those with the t(8;21)(q22;q22) karyotype, 120 patients (35.50%) showed loss of a sex chromosome, including 80 (23.67%) with loss of the Y chromosome and 40 (11.83%) with loss of the X chromosome. Detailed patient characteristics are summarised in Table 1.

**Table 1:** Characteristics of 338 acute myeloid leukaemia (AML) with t(8;21) patients

Variable	Number (%)/median (range)
Sex	
Male	173 (51.18%)
Female	165 (48.82%)
Median age (years)	44 (5–82)
WBC at diagnosis, 10^9^ L^−1^	
< 20	248 (73.37%)
≥ 20	67 (19.82%)
No information	23 (6.80%)
FAB classification	
Did not exceed the 20% blasts without FAB classification	20 (5.92%)
FAB‐M0/1/2	208 (61.54%)
M0	1 (0.30%)
M1	3 (0.89%)
M2	204 (60.36%)
FAB‐M4/5	102 (30.18%)
M4	19 (5.62%)
M5	83 (24.56%)
Cell source re‐classification based on immunophenotypic stratification	
Immuno‐Prim	160 (47.34%)
MPP	68 (20.12%)
LMPP	49 (14.50%)
CMP	43 (12.72%)
Immuno‐Mono	119 (35.21%)
GMP	84 (24.85%)
GP/MP	35 (10.36%)
Karyotype	
t(8;21)(q22;q22)	244 (72.19%)
t(8;21)(q22;q22), −Y	80 (23.67%)
t(8;21)(q22;q22), −X	40 (11.83%)
Normal karyotype	32 (9.47%)
Complex karyotype	21 (6.21%)
Others	41 (12.13%)
Gene mutations	
KIT	136 (40.24%)
FLT3	56 (16.57%)
NRAS	42 (12.43%)
TET2	40 (11.83%)
ASXL1	37 (10.95%)
Median follow‐up (days)	874 (61–3566)

### Immunophenotypic definition of Immuno‐Prim and Immuno‐Mono patients

**Figure 2:**
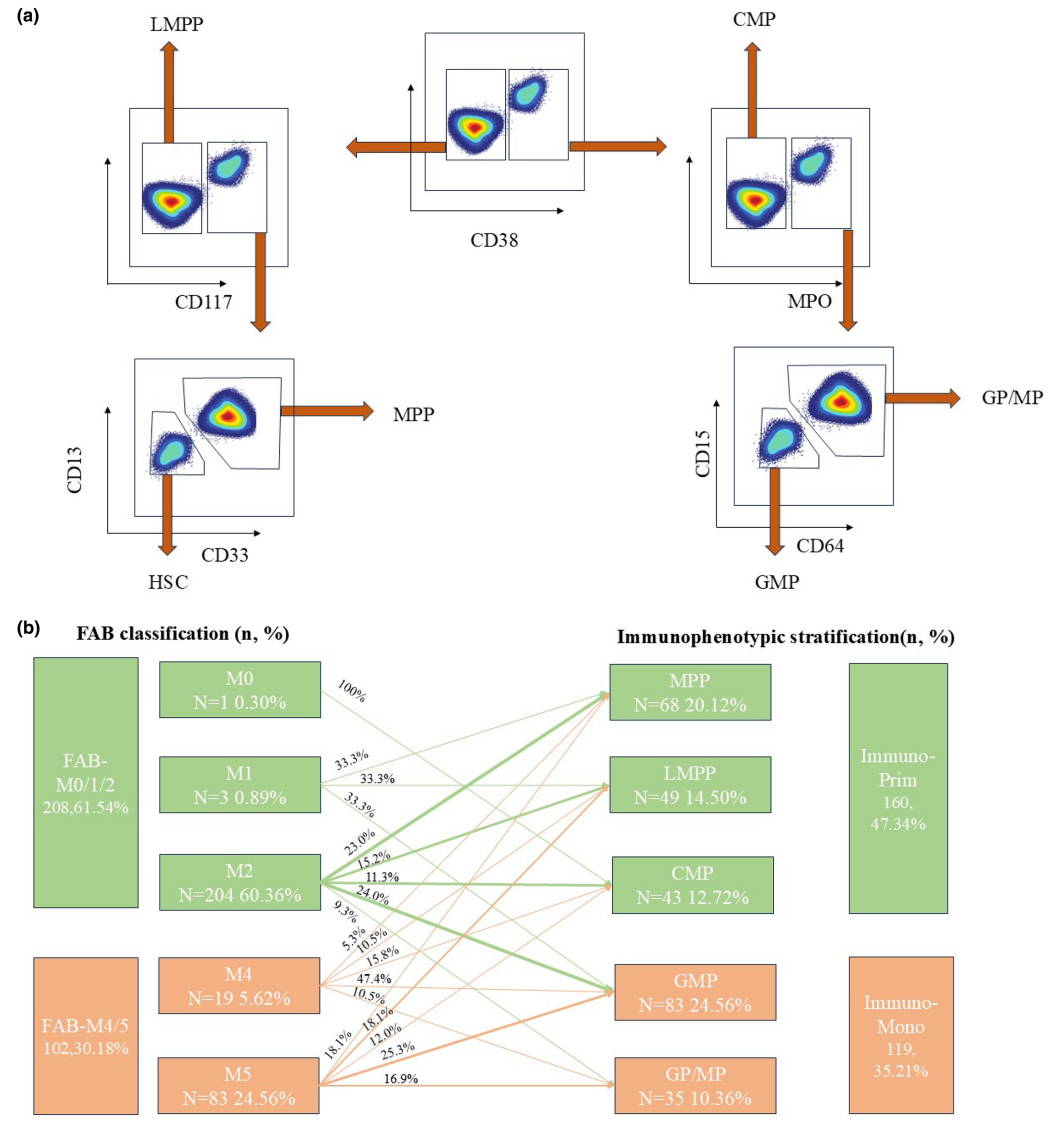
Immunophenotypic definition of Immuno‐Prim and Immuno‐Mono patients. **(a)** The definition of immunophenotypic primitive (Immuno‐Prim) group and monocytic (Immuno‐Mono) group based on immunophenotypic markers in primary diagnosis sample with flow cytometry methods. **(b)** Sankey diagram comparing French‐American‐British (FAB) and Immuno categories.

According to the traditional FAB classification, one patient (0.30%) was classified as M0, 3 (0.89%) as M1, 204 (60.36%) as M2, 19 (5.62%) as M4 and 83 (24.56%) as M5. Therefore, 208 patients (61.54%) were grouped into the FAB‐M0/1/2 category and 102 patients (30.18%) into the FAB‐M4/M5 group. Immunophenotyping data from diagnostic bone marrow samples were available for 279 patients. As detailed in the Methods section, immunophenotypic stratification was performed in a stepwise manner based on flow cytometric expression of specific markers. First, the expression of CD38 was assessed. If CD38 was positive, the expression of MPO was then evaluated. CD38‐positive but MPO‐negative cells were classified as CMP. If both CD38 and MPO were positive, CD64 and CD15 were assessed. Cells expressing either CD64 or CD15 were categorised as GP/MP, whereas those lacking both markers were defined to be GMP. If CD38 was negative, the expression of CD117 was then assessed. CD38‐negative and CD117‐negative cells were identified as lymphoid‐primed multi‐potent progenitor (LMPP). If CD117 was positive, the expression of CD13 or CD33 was examined. If either of these markers was positive, the cells were defined to be MPP; otherwise, they were classified as HSC, as illustrated in Figure 2a. Based on this immunophenotypic stratification, 68 patients (20.12%) were classified as MPP, 49 (14.50%) as LMPP, 43 (12.72%) as CMP, 84 (24.85%) as GMP and 35 (10.36%) as GP/MP. Consequently, 160 patients (47.34%) were assigned to the Immuno‐Prim group (comprising HSC, MPP, LMPP and CMP), while 119 patients (35.21%) were assigned to the Immuno‐Mono group (comprising GMP and GP/MP), as shown in Figure 2b. The characteristics of patients in the Immuno‐Prim and Immuno‐Mono groups are summarised in Supplementary table 2.

### The landscape of gene mutations in AML with t(8;21) patients

**Figure 3:**
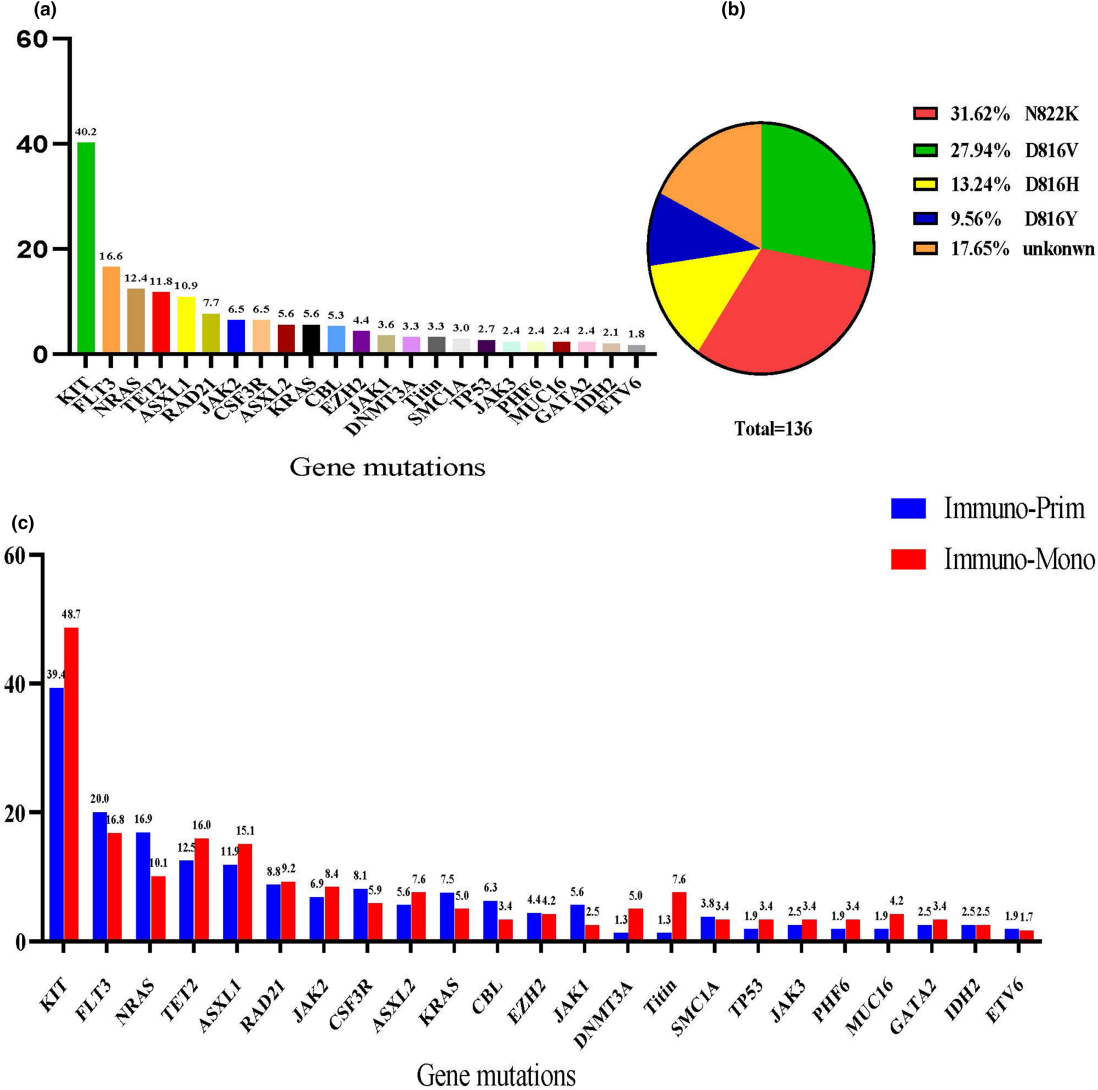
Landscape of gene mutations in acute myeloid leukaemia (AML) with t(8;21) patients. **(a)** Most gene mutations in the entire group. **(b)** Most frequencies of KIT gene location. **(c)** Most gene mutations between Immuno‐Prim and Immuno‐Mono groups based on immunophenotypic stratification.

**Figure 4:**
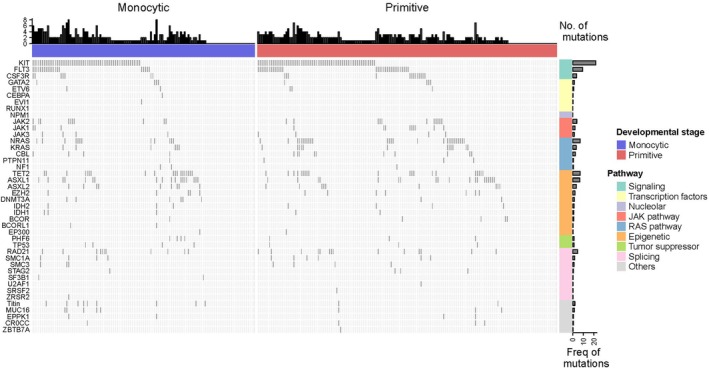
Landscape of gene mutations in acute myeloid leukaemia (AML) with t(8;21) patients between Immuno‐Prim and Immuno‐Mono groups based on immunophenotypic stratification.

Gene mutation data at diagnosis were available for 312 patients. The most frequently observed mutations across the cohort were in KIT (40.24%), FLT3 (16.57%), NRAS (12.43%), TET2 (11.83%), ASXL1 (10.95%), RAD21 (7.69%), JAK2 (6.51%), CSF3R (6.51%), ASXL2 (5.62%) and KRAS (5.62%) (Figure 3a). A total of 136 patients harboured KIT mutations. The most common KIT variants were N822K (31.62%), D816V (27.94%), D816H (13.24%) and D816Y (9.56%) (Figure 3b). The frequency of major gene mutations was generally comparable between the Immuno‐Prim and Immuno‐Mono groups (Figure 3c). However, *Titin* mutations were significantly more frequent in the Immuno‐Mono group than in the Immuno‐Prim group (7.6% vs. 1.3%, *P* = 0.018). No other gene mutations showed significant differences between the two immunophenotypic groups (Supplementary table 3, Figure 4).

### Impact of differentiation stages on results of induction chemotherapy

Patients initially received induction chemotherapy with one of the following regimens: idarubicin and cytarabine (IA), daunorubicin and cytarabine (DA), homoharringtonine, cytarabine and aclarubicin (HAA), or venetoclax‐based therapy. The CR rates were 73.83% (110/149) for IA, 80.85% (38/47) for DA, 71.05% (27/38) for HAA and 60.27% (44/73) for venetoclax‐based regimens (*P* = 0.075). When stratified by immunophenotype, the CR rates in the Immuno‐Prim group were 69.39% (34/49) for IA, 84.00% (21/25) for DA, 78.57% (11/14) for HAA and 61.40% (35/57) for venetoclax‐based regimens (*P* = 0.187). In the Immuno‐Mono group, CR rates were 74.14% (43/58) for IA, 80.00% (16/20) for DA, 65.00% (13/20) for HAA and 56.25% (9/16) for venetoclax‐based regimens (*P* = 0.375). The venetoclax and azacitidine (VA) combination regimen was used as induction therapy in a separate cohort of 26 patients. The overall CR rate for the VA regimen was 26.92% (7/26). When divided by immunophenotypic group, the CR rate was 26.32% (5/19) in the Immuno‐Prim group and 28.57% (2/7) in the Immuno‐Mono group (*P* = 1.000) (Supplementary table 4). A total of 95 patients relapsed during chemotherapy. Among these, reinduction therapy achieved CR rates of 68.75% (11/16) with cladribine/fludarabine‐based regimens, 57.14% (12/21) with HAA and 28.57% (4/14) with venetoclax‐based regimens (*P* = 0.078).

### Impact of stages of differentiation and gene mutations on clinical outcomes in the entire cohort

**Figure 5:**
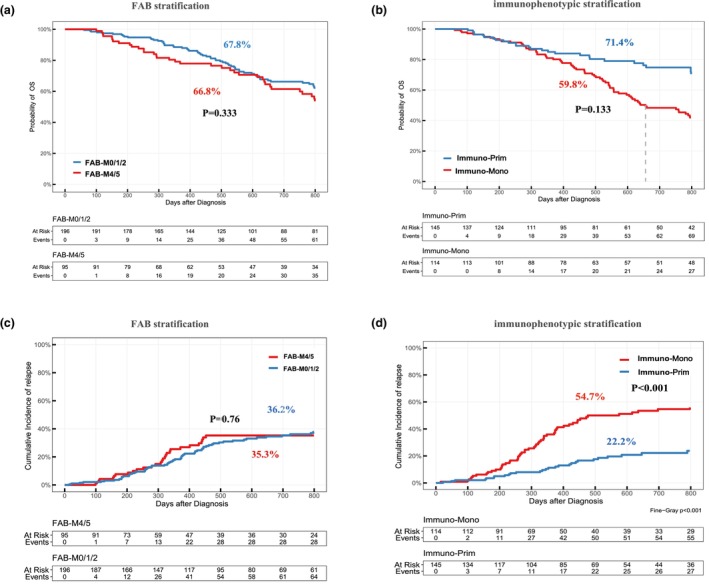
Impact of differentiation stages on clinical outcomes in the entire cohort. **(a)** The difference in overall survival (OS) between FAB‐M0/1/2 and FAB‐M4/5 groups based on FAB stratification. **(b)** The difference in OS between Immuno‐Prim and Immuno‐Mono groups based on immunophenotypic stratification. **(c)** The difference in cumulative incidence of relapse (CIR) between FAB‐M0/1/2 and FAB‐M4/5 groups based on FAB stratification. **(d)** The difference in CIR between Immuno‐Prim and Immuno‐Mono groups based on immunophenotypic stratification. FAB, French‐American‐British.

There were no significant differences in 2‐year overall survival (OS) or 2‐year cumulative incidence of relapse (CIR) between the FAB‐M0/1/2 and FAB‐M4/5 groups, based on FAB classification (2‐year OS 67.8% vs. 66.8%, *P* = 0.333; 2‐year CIR 36.2% vs. 35.3%, *P* = 0.760; Figure 5a and c). However, when using immunophenotypic stratification, the Immuno‐Mono group showed a trend towards lower 2‐year OS and a significantly higher 2‐year CIR compared to the Immuno‐Prim group (2‐year OS 59.8% vs. 71.4%, *P* = 0.133; 2‐year CIR 54.7% vs. 22.2%, *P* < 0.001; Figure 5b and d). Patients with KIT mutations had significantly worse 2‐year OS and higher 2‐year CIR than those without KIT mutations (2‐year OS 50.8% vs. 80.2%, *P* < 0.001; 2‐year CIR 44.9% vs. 29.3%, *P* = 0.008; Supplementary figure 1a and e). In contrast, mutations in FLT3 and NRAS, as well as the loss of X or Y chromosomes, had no significant impact on OS or CIR in the overall cohort (Supplementary table 5; Supplementary figure 1b–d, f–h). Stratified analysis revealed that the Immuno‐Mono group with KIT mutations and the Immuno‐Prim group with KIT mutations had significantly lower 2‐year OS than their counterparts without KIT mutations (Immuno‐Mono with vs. without KIT 42.9% vs. 74.5%, *P* < 0.001; Immuno‐Prim with vs. without KIT 52.5% vs. 82.8%, *P* < 0.001; Supplementary figure 2a). Similarly, the Immuno‐Mono group with FLT3 mutations had significantly worse 2‐year OS compared to the Immuno‐Prim group with FLT3 mutations, the Immuno‐Mono group without FLT3 mutations and the Immuno‐Prim group without FLT3 mutations (30.8% vs. 64.4% vs. 65.0% vs. 73.4%, *P* < 0.001; Supplementary figure 2b). Regarding relapse, both the Immuno‐Mono group with and without KIT mutations had significantly higher 2‐year CIR than the Immuno‐Prim group with and without KIT mutations, respectively (56.0% vs. 52.6% vs. 35.6% vs. 14.4%, *P* < 0.001; Supplementary figure 2c). A similar pattern was observed for FLT3 mutations: the Immuno‐Mono group with and without FLT3 mutations had significantly higher 2‐year CIR than the Immuno‐Prim group with and without FLT3 mutations (64.2% vs. 52.8% vs. 21.3% vs. 22.4%, *P* < 0.001; Supplementary figure 2d). Univariate analysis results for AML patients with t(8;21) are summarised in Supplementary table 5.

### Impact of differentiation stages and gene mutations on clinical outcomes in the allo‐HSCT cohort

**Figure 6:**
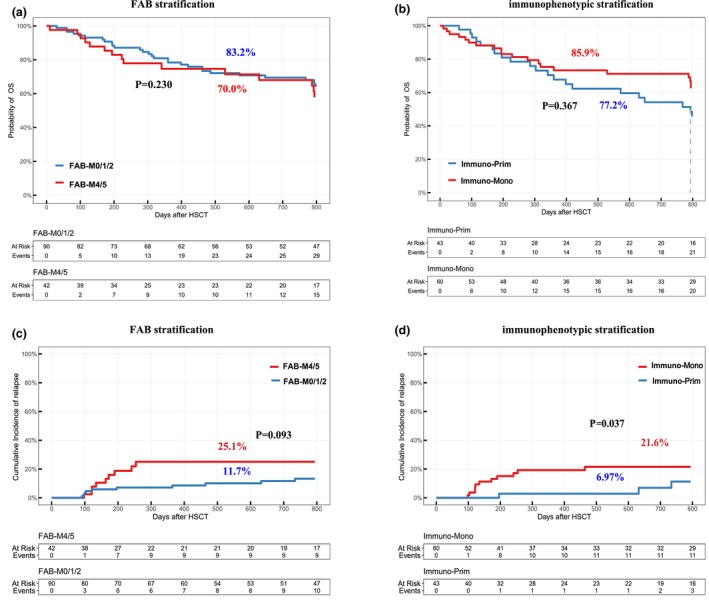
Impact of differentiation stages on clinical outcomes in the allo‐HSCT group. **(a)** The difference in overall survival (OS) between FAB‐M0/1/2 and FAB‐M4/5 groups based on FAB stratification. **(b)** The difference in OS between Immuno‐Prim and Immuno‐Mono groups based on immunophenotypic stratification. **(c)** The difference in cumulative incidence of relapse (CIR) between FAB‐M0/1/2 and FAB‐M4/5 groups based on FAB stratification. **(d)** The difference in CIR between Immuno‐Prim and Immuno‐Mono groups based on immunophenotypic stratification. FAB, French‐American‐British.

Among the 141 patients who underwent allo‐HSCT in our cohort, 34.8% (49/141) were MRD‐positive prior to transplantation. MRD positivity rates did not significantly differ between FAB subgroups, with 34.4% (31/90) in the FAB‐M0/M1/M2 group and 35.7% (15/42) in the FAB‐M4/M5 group (*P* = 0.887). When stratified by immunophenotype, MRD positivity was observed in 27.9% (12/43) of Immuno‐Prim patients and 41.7% (25/60) of Immuno‐Mono patients; however, this difference was not statistically significant (*P* = 0.151). With regard to transplant timing, 70.2% (99/141) of patients underwent allo‐HSCT in CR1, while 29.8% (42/141) were transplanted in CR2 following relapse. The proportion of CR2 transplants was similar between FAB subgroups (30.0% [27/90] in FAB‐M0/M1/M2 vs. 28.6% [12/42] in FAB‐M4/M5; *P* = 0.867). However, a significantly higher proportion of Immuno‐Mono patients received transplantation in CR2 than Immuno‐Prim patients (41.7% [25/60] vs. 16.3% [7/43]; *P* = 0.006). In the allo‐HSCT cohort, there were no significant differences in 2‐year OS and 2‐year CIR between the FAB‐M0/1/2 and FAB‐M4/5 groups (2‐year OS 83.2% vs. 70.0%, *P* = 0.230; 2‐year CIR 11.7% vs. 25.1%, *P* = 0.093; Figure 6a and c). However, based on immunophenotypic stratification, the Immuno‐Mono group demonstrated a trend towards lower 2‐year OS and significantly higher 2‐year CIR than the Immuno‐Prim group (2‐year OS 77.2% vs. 85.9%, *P* = 0.367; 2‐year CIR 21.6% vs. 6.97%, *P* = 0.037; Figure 6b and d). Mutations in KIT, FLT3 and NRAS, as well as the loss of X or Y chromosomes, did not significantly impact either OS or CIR among patients in the transplant cohort (Supplementary figure 3a–h). However, the differentiation stage of leukaemia cells remained an important determinant of post‐transplant outcomes. Specifically, both the Immuno‐Mono group with and without KIT mutations had significantly higher 2‐year CIR than the Immuno‐Prim group with and without KIT mutations, respectively (20.10% vs. 19.80% vs. 6.27% vs. 9.52%; *P* = 0.024; Supplementary figure 4c). Similarly, the Immuno‐Mono group with and without FLT3 mutations had significantly higher 2‐year CIR than the Immuno‐Prim group with and without FLT3 mutations (55.6% vs. 14.5% vs. 13.7% vs. 6.84%; *P* < 0.01; Supplementary figure 4d). Additionally, patients who were MRD‐positive prior to allo‐HSCT had significantly lower 2‐year OS and higher 2‐year CIR compared to MRD‐negative patients. Patients transplanted in CR2 also exhibited worse outcomes, with lower 2‐year OS and higher 2‐year CIR than those transplanted in CR1 (Supplementary table 4).

### Multivariate analysis confirmed developmental stage correlates closely with outcomes

Based on the results of univariate analysis and the reported potential impact of NRAS mutations and sex chromosome loss (loss of X or Y), we included the following variables in a multivariate analysis for OS and CIR in the entire cohort: KIT (mutant vs. wild‐type), FLT3 (mutant vs. wild‐type), NRAS (mutant vs. wild‐type), patient age (≥ 44 vs. < 44 years), WBC at diagnosis (≥ 20 vs. < 20 × 10^9^ L^−1^), immunophenotypic subgroup (Immuno‐Mono vs. Immuno‐Prim) and loss of X or Y chromosome (yes vs. no). KIT mutation, older age and elevated WBC at diagnosis were identified as independent predictors of 2‐year OS in the overall cohort (KIT HR = 1.871, 95% CI 0.818–4.281 *P* < 0.001; age HR = 0.462, 95% CI 0.289–0.740, *P* = 0.001; WBC HR = 0.473, 95% CI 0.288–0.776, *P* = 0.003). Age and differentiation stage (Immuno‐Mono vs. Immuno‐Prim) were independent predictors of 2‐year CIR (age HR = 6.339, 95% CI 1.916–20.971, *P* = 0.003; Immuno‐Mono vs. Immuno‐Prim HR = 3.060, 95% CI 1.129–8.270, *P* = 0.018). There was a significant association between pre‐transplant MRD status and the remission status at the time of transplantation. Among MRD‐negative patients, only 19.6% underwent allo‐HSCT in second remission, compared to 49.0% of MRD‐positive patients (*P* < 0.001). Given its importance as a prognostic factor, we included pre‐HSCT MRD status in the multivariate analysis for OS and CIR within the transplant cohort. In this subgroup, WBC at diagnosis was the independent predictor of 2‐year OS (HR = 0.272, 95% CI 0.102–0.724, *P* = 0.009). Immunophenotypic classification (Immuno‐Mono vs. Immuno‐Prim) influenced the 2‐year CIR in the transplant cohort (HR = 0.151, 95% CI 0.017–1.330, *P* = 0.088), although the result did not reach statistical significance. Full multivariate results are presented in Table 2.

**Table 2:** Multivariable analysis of clinical outcomes of all patients

Variable	Entire group				Transplant group			
	OS		CIR		OS		CIR	
	HR (95% CI)	*P*	HR (95% CI)	*P*	HR (95% CI)	*P*	HR (95% CI)	*P*
KIT (mut vs. wt)	0.389 (0.244–0.621)	**< 0.001**	1.871 (0.818–4.281)	0.140	0.735 (0.282–1.912)	0.528	1.872 (0.378–9.26)	0.440
FLT3 (mut vs. wt)	0.810 (0.471–1.393)	0.446	0.793 (0.311–2.026)	0.630	0.507 (0.173–1.484)	0.215	3.092 (0.517–18.490)	0.220
NRAS (mut vs. wt)	1.255 (0.624–2.525)	0.523	1.889 (0.705–5.063)	0.210	0.742 (0.236–2.333)	0.610	2.024 (0.260–15.790)	0.500
Loss of X or Y (yes vs. no)	0.942 (0585–1.518)	0.807	1.013 (0.427–2.402)	0.980	1.065 (0.393–2.884)	0.901	1.210 (0.206–7.120)	0.830
Age (≥ 44 vs. < 44), y	0.462 (0.289–0.740)	**0.001**	6.339 (1.916–20.971)	**0.003**	0.578 (0.213–1.567)	0.281	4.428 (0.712–27.550)	0.110
WBC (≥ 20 vs. < 20), ×109/L	0.473 (0.288–0.776)	**0.003**	2.273 (0.748–6908)	0.150	0.272 (0.102–0.724)	**0.009**	1.528 (0.169–2.530)	0.540
Immuno‐Mono vs. Immuno‐Prim	0.787 (0.502–1.232)	0.294	3.060 (0.129–0.827)	**0.018**	0.550 (0.197–1.534)	0.253	0.151 (0.017–1.330)	**0.088**
Pre‐HSCT MRD + vs. −					0.523 (0.205–1.392)	0.173	1.209 (0.180–8.130)	0.850

Values in bold are significant (*P* < 0.01).

## Discussion

Although the t(8;21) translocation encodes an oncoprotein that, in combination with co‐occurring mutations, drives leukaemogenesis, AML with t(8;21)/RUNX1–RUNX1T1 exhibits distinct developmental stages of leukaemic cells, as revealed by immunophenotypic stratification. Our study demonstrates that these differentiation hierarchies significantly influence clinical outcomes in patients with this subtype. Specifically, monocytic AML was associated with a higher CIR and poorer prognosis compared to primitive AML. Among all groups, monocytic AML with KIT mutations exhibited the highest CIR, both in the overall cohort and the transplant subgroup. While allo‐HSCT was able to mitigate the adverse impact of KIT mutations, it did not fully overcome the negative influence of leukaemic differentiation stage. To the best of our knowledge, this is the first study to systematically characterise the heterogeneity of AML with t(8;21)/RUNX1–RUNX1T1 based on developmental stage subgroups.

Interestingly, our findings show that several prognostic factors were significantly associated with CIR but not with OS, suggesting that modern salvage therapies may have effectively reduced mortality after relapse. However, we hypothesise that with longer follow‐up, differences in OS may become more apparent as clinical outcomes continue to mature. IA‐based induction therapy remains the standard of care for fit patients with AML, while the VA regimen is commonly used for older or unfit patients.^1^ In our study, the VA regimen resulted in the lowest CR rates, both in newly diagnosed and relapsed patients. This may be as a result of relatively low BCL‐2 expression in AML with t(8;21)/RUNX1–RUNX1T1.^16^ Previous studies have similarly reported that patients with the AML1–ETO fusion transcript respond poorly to frontline VEN/HMA therapy compared to other AML subtypes treated with the same regimen.^17^, ^18^, ^19^ Our data also demonstrate that patient age significantly influences both OS and CIR. Beyond age‐related factors, such as comorbidities and performance status, the poor response to VEN/HMA therapy in older patients with AML1–ETO likely contributes to the low CR rates and high relapse rates observed in this subgroup.^20^

The genomic landscape of AML with t(8;21)/RUNX1–RUNX1T1 was consistent with previous reports.^10^, ^11^ KIT is a key kinase gene for which no effective targeted therapies currently exist. The prognostic impact of KIT mutations in AML with t(8;21)/RUNX1–RUNX1T1 has been well documented in numerous studies.^10^, ^11^, ^21^, ^22^ The 2017 ELN guidelines discussed RUNX1–RUNX1T1 with concurrent KIT mutations as being associated with poor prognosis. Activating c‐KIT mutations contribute to leukaemogenesis by enhancing DNA repair mechanisms and inhibiting apoptosis, offering a potential mechanistic explanation for the observed chemoresistance in t(8;21) AML with cooperating c‐KIT mutations.^23^ Additionally, c‐KIT activating mutations have been shown to confer resistance to PARP inhibitors in AML1–ETO–positive leukaemias.^24^ It is important to acknowledge recent advances led by large‐scale collaborative consortia. Notably, the HARMONY Alliance and similar initiatives have developed machine learning–based prognostic models that incorporate the combined effects of multiple recurrent AML mutations on survival outcomes.^25^, ^26^ These integrative approaches may offer a more comprehensive understanding of the complex genetic interactions in AML than traditional single‐variable analyses.

Acute myeloid leukaemia cells exist across a spectrum of differentiation states, each exhibiting distinct tumorigenic potential and responses to chemotherapy.^7^, ^27^ The heterogeneity of AML leukaemia stem cells (LSCs) has direct clinical implications.^8^, ^27^, ^28^ In particular, monocytic AML cells lose expression of BCL2, the primary target of venetoclax, and instead rely on MCL1 to support oxidative phosphorylation and survival. This shift contributes to resistance against venetoclax‐based therapies.^8^ Conventional chemotherapy regimens show limited efficacy across the full range of LSC subpopulations, as their therapeutic targets fail to address the underlying molecular heterogeneity of these subtypes. This biological limitation contributes to persistent minimal residual disease and increases the risk of relapse.^28^ There is a clear need to understand how heterogeneity in developmental stage impacts treatment response within AML subtypes sharing the same fusion gene. In our study, we observed significant heterogeneity in immunophenotypes among RUNX1–RUNX1T1 AML patients, which translated into distinct clinical outcomes. Patients with monocytic AML had lower OS and higher CIR compared to those with primitive AML. Notably, monocytic RUNX1–RUNX1T1 AML patients harbouring KIT mutations had the poorest prognosis in our cohort. This suggests that monocytic RUNX1–RUNX1T1 AML with KIT mutations may warrant re‐classification into intermediate‐ or high‐risk categories in future stratification schemes. While genetic diversity likely contributes to LSC heterogeneity,^6^, ^9^ we did not observe significant differences in gene mutation profiles between immunophenotypic subgroups in our RUNX1–RUNX1T1 AML cohort. The limitation may lie in the scope of our 92‐gene panel, which may not capture the full extent of genetic variability. Transcriptomic differences between subclones may help explain the variation in tumorigenic behaviour and therapy response, underscoring the need for further investigation. Future studies should aim to isolate and characterise AML cells at different developmental stages within the RUNX1–RUNX1T1 subtype to better understand their biological and therapeutic heterogeneity. In our cohort, monocytic AML cells were resistant to venetoclax‐based therapy, with the lowest CR rates observed in patients treated with the VA regimen. However, cladribine has shown sensitivity against monocytic LSCs by targeting purine metabolism,^28^ and homoharringtonine‐based regimens have proven effective in RUNX1–RUNX1T1 AML, both in previous studies and in our findings.^29^, ^30^, ^31^ These data suggest that cladribine‐ and homoharringtonine‐based therapies may be promising treatment options for patients with t(8;21) AML.

Haematopoietic stem cell transplantation is a potentially curative approach for AML patients, largely because of the sustained GVL effect observed *in vivo*. Pre‐MRD is an important indicator of tumor burden and a key predictor of post‐transplant relapse risk.^32^ In our cohort, pre‐MRD positivity was observed in 27.9% (12/43) of Immuno‐Prim patients and 42.6% (25/60) of Immuno‐Mono patients (*P* = 0.092). In the transplant setting, there was no significant difference in OS or CIR between KIT‐mutant and KIT wild‐type patients. However, the Immuno‐Mono group exhibited a significantly higher CIR than the Immuno‐Prim group following transplantation. These findings suggest that while HSCT can mitigate the adverse impact of KIT mutations, it does not fully overcome the influence of immunophenotypic differences among LSC populations on clinical outcomes in RUNX1–RUNX1T1 AML. The durable disease‐free status observed in many post‐transplant patients is attributed to immune surveillance mediated by the GVL effect.^33^, ^34^ The increased relapse risk observed in the Immuno‐Mono group may reflect immune evasion mechanisms that weaken the GVL response.

Our study has several limitations that should be acknowledged. Chemotherapy regimens varied among patients based on age and comorbidities. The sample size, particularly in subgroup analyses, was limited. Further studies are warranted to clarify the clinical implications of developmental stage heterogeneity in AML patients with t(8;21)/RUNX1–RUNX1T1. Additionally, the restricted mutation panel used in this study may have limited our ability to detect genetic differences between groups. As a retrospective clinical study, we were unable to include HLA expression data on leukaemic blasts, which may have affected immune interactions and transplant outcomes. Moreover, our dataset lacked complete information on the use of FLT3 inhibitors, which may have acted as a confounding variable. The question of FLT3 inhibitor efficacy in RUNX1–RUNX1T1 AML remains unresolved and represents an important avenue for future investigation. Finally, the underlying mechanisms driving the higher relapse rates in monocytic RUNX1–RUNX1T1 AML remain unclear and should be a focus of future research.

## Conclusion

Our study demonstrates that AML with t(8;21)/RUNX1–RUNX1T1 is arrested at specific stages of myeloid differentiation, which significantly correlates with genetic lesions, clinical presentation, chemosensitivity, treatment response and overall outcomes.

## Methods

### Patients

We enrolled patients diagnosed with AML characterised by the recurrent genetic subtype t(8;21)/RUNX1–RUNX1T1 who were treated at the First Affiliated Hospital, Zhejiang University School of Medicine; the Affiliated Jinhua Hospital, Zhejiang University School of Medicine; and the Affiliated Jinhua Hospital of Wenzhou Medical University between January 2018 and August 2023. All cases of t(8;21)(q22;q22) were molecularly confirmed by quantitative reverse transcription–polymerase chain reaction (RT‐qPCR) detection of the *RUNX1::RUNX1T1* fusion transcript. This diagnostic approach is consistent with the recommendations of the 2022 ELN for core‐binding factor AML. A total of 338 patients with t(8;21) AML were included in the study. Detailed flow cytometric immunophenotyping at initial diagnosis was available for 279 of these patients. All participants provided written informed consent for the potential use of their clinical information in research prior to treatment initiation. The study was approved by the ethics review committees of the First Affiliated Hospital of Zhejiang University School of Medicine, the Affiliated Jinhua Hospital of Zhejiang University School of Medicine and the Affiliated Jinhua Hospital of Wenzhou Medical University. The study was conducted in accordance with the principles outlined in the Declaration of Helsinki.

### Definition of Immuno‐Prim and Immuno‐Mono patients

According to the traditional morphological FAB classification, M0, M1 and M2 cases were grouped as FAB‐M0/1/2, while M4 and M5 cases were grouped as FAB‐M4/5. In parallel, AML blasts were also classified into immunophenotypic primitive (Immuno‐Prim) and monocytic (Immuno‐Mono) groups based on the expression of CD34, CD117, CD13, CD33, CD38, MPO, CD11b, CD15 and CD64 in diagnostic samples using flow cytometry. As previously described, CD38^+^/MPO^+^/CD64^+^ or CD15^+^ cells were defined to be granulocyte progenitor/monocyte progenitor (GP/MP), CD38^+^/MPO^+^/CD13^+^ or CD33^+^/CD64^−^ as granulocyte‐monocyte progenitor (GMP), CD38^+^/MPO^−^/CD117^+^ or CD13^+^/CD64^−^ as common myeloid progenitor (CMP), CD38^−^/MPO^−^/CD117^−^/CD13^+^ or CD33^+^ as LMPP, CD38^−^/MPO^−^/CD117^+^/CD13^+^ or CD33^+^ as multi‐potent progenitor (MPP), and CD38^−^/MPO^−^/CD117^+^/CD13^−^/CD33^−^ as haematopoietic stem cell (HSC). Based on this immunophenotypic stratification,^15^, ^35^, ^36^ HSC, MPP, LMPP and CMP were classified as Immuno‐Prim, and GMP and GP/MP as Immuno‐Mono. Leukaemia‐associated immunophenotypes (LAIPs) were detected using 10‐colour flow cytometry (FCM), and methods were standardised between laboratories according to consensus guidelines for clinical application.^37^ A 0.1% threshold was used to distinguish positive from negative expression.^38^ Complete remission was defined per ELN 2022 criteria to be < 5% bone marrow blasts with peripheral blood recovery (ANC ≥ 1.0 × 10^9^ L^−1^ and platelets ≥ 100 × 10^9^ L^−1^) and no extramedullary disease. Relapse was defined to be ≥ 5% blasts in the bone marrow, recurrence of blasts in peripheral blood or development of extramedullary disease. Measurable residual disease (MRD) prior to HSCT was provisionally defined to be ≥ 0.2% variant allele frequency (VAF) as detected by digital PCR (dPCR) on genomic DNA.^38^

### Next‐generation sequencing

All patients underwent next‐generation sequencing (NGS) for mutations in a panel of 92 myeloid‐related genes using high‐throughput sequencing performed in‐house (Supplementary table 1). A VAF threshold of ≥ 1% was considered positive for mutation detection. Gene mutation data were unavailable for 46 patients because of the retrospective nature of the study.

### HSCT procedure

All patients received mobilised peripheral blood stem cells as the graft source. Conditioning regimens and graft‐versus‐host disease (GVHD) prophylaxis were administered as previously described.^32^, ^39^ GVHD prophylaxis included anti‐thymocyte globulin (ATG), cyclosporin A, methotrexate and low‐dose mycophenolate mofetil, in accordance with previously established protocols.^32^, ^40^ Immunosuppressive agents were tapered and discontinued 6–9 months after HSCT in patients who showed no signs of GVHD.

### Statistics

Data were assessed for normal distribution, and subsequent statistical tests were chosen accordingly. Continuous variables were analysed using Student's *t*‐test or the Mann–Whitney *U*‐test, while categorical variables were evaluated using Pearson's chi‐square test or Fisher's exact test. Overall survival was estimated using the Kaplan–Meier method. Relapse was treated as a competing event for non‐relapse mortality (NRM). The CIR was calculated using the competing risk method as described by Gooley and Scrucca,^41^, ^42^ allowing for comparisons based on relevant competing events.^43^ Cox proportional hazards regression models were used to assess factors associated with OS. In contrast, Fine and Gray's subdistribution hazard model was used to evaluate relapse, accounting for potential confounders in multivariate analysis.^44^ All statistical analyses were conducted using SPSS version 22.0 (IBM Corp., Armonk, NY) and R version 3.6.1 (http://www.r‐project.org). A two‐sided *P*‐value < 0.05 was considered statistically significant.

## Author contributions

**Yibo Wu:** Data curation; funding acquisition; resources; writing – original draft; writing – review and editing. **Xiaolin Yuan:** Data curation; formal analysis; investigation; writing – review and editing. **Xiaoyu Lai:** Conceptualization; investigation. **Lizhen Liu:** Conceptualization; data curation. **Yue Liang:** Data curation; methodology; software. **Lihong Ni:** Investigation. **Luxin Yang:** Investigation; methodology; supervision. **Shanshan Hu:** Conceptualization; investigation. **Jimin Shi:** Conceptualization; data curation; investigation. **Jian Yu:** Data curation; investigation; methodology. **Yanmin Zhao:** Data curation; investigation. **Weiyan Zheng:** Conceptualization; investigation. **Jie Sun:** Conceptualization; data curation. **Yuanyuan Zhu:** Conceptualization; data curation. **Wenjun Wu:** Conceptualization; investigation. **Zhen Cai:** Supervision; visualization. **He Huang:** Resources; supervision; visualization. **Shanshan Pei:** Methodology; project administration. **Yi Luo:** Funding acquisition; project administration; resources; supervision; validation.

## Conflict of interest

The authors declare no conflict of interest.

## Supporting information


Supplementary figure 1
Supplementary figure 2
Supplementary figure 3
Supplementary figure 4
Supplementary table 1
Supplementary table 2
Supplementary table 3
Supplementary table 4
Supplementary table 5

## Data Availability

The data supporting the findings of this study are available from the corresponding author upon reasonable request.
