# Supplementary material for: Cellular hierarchy for understanding heterogeneity of acute myeloid leukaemia with t(8;21)/RUNX1‐RUNX1T1
Source: Clin Transl Immunology. 2025 Jul 2;14(7):e70042. doi: 10.1002/cti2.70042 (PMC12221812; doi:10.1002/cti2.70042)
Supplement: Supplementary file 1 — Supplementary figure 1 Supplementary figure 2 Supplementary figure 3 Supplementary figure 4 Supplementary table 1 Supplementary table 2 Supplementary table 3 Supplementary table 4 Supplementary table 5 [file CTI2-14-e70042-s001.docx]

**SUPPORTING INFORMATION**


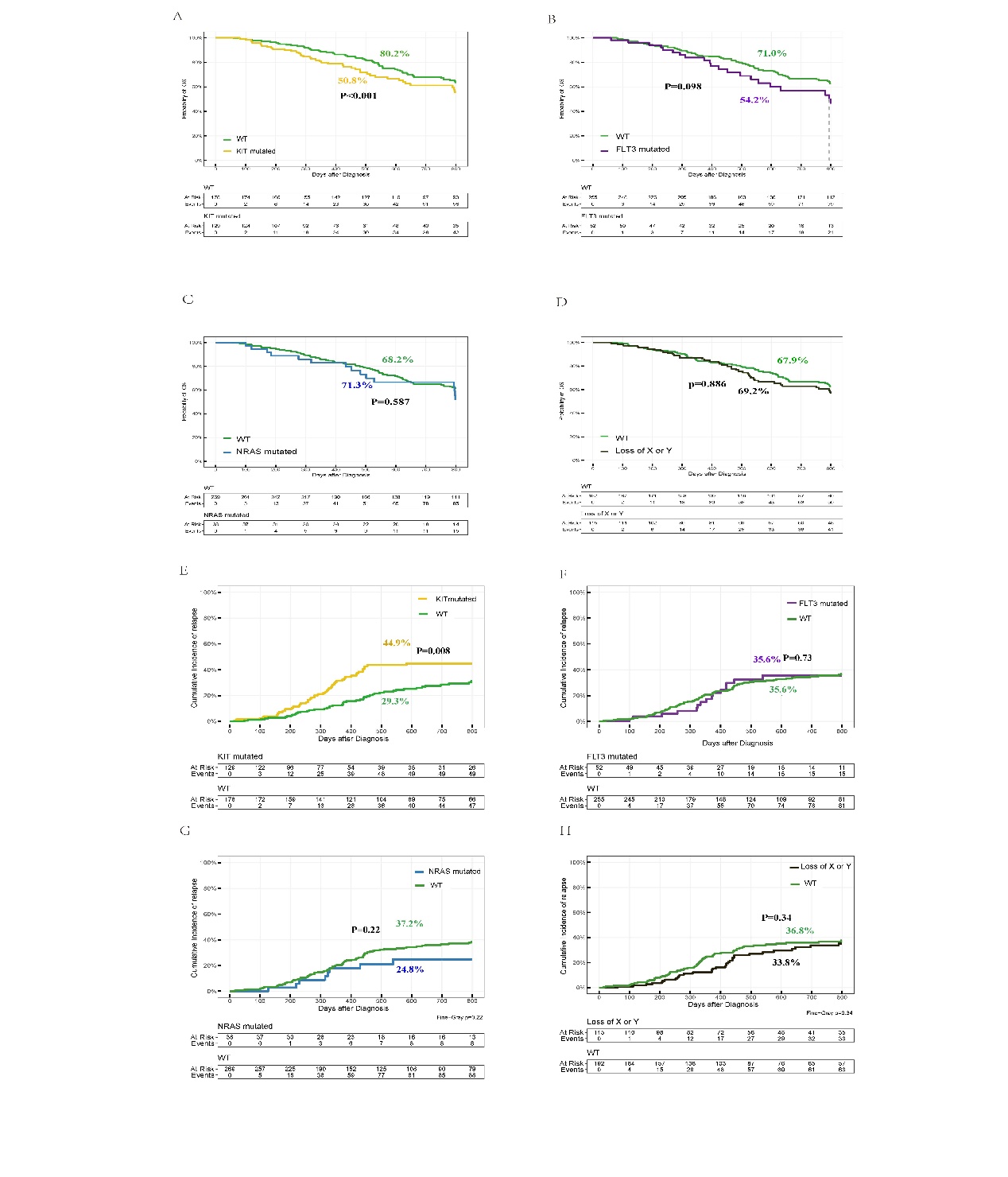


**Supplementary figure 1. Impact of gene mutations on clinical outcomes in the entire cohort.**

**(a)** The difference of overall survival in patients with or without KIT mutation. **(b)** The difference of overall survival in patients with or without FLT3 mutation. **(c)** The difference of overall survival in patients with or without NRAS mutation. **(d)** The difference of overall survival in patients with or without loss of X or Y. **(e)** the difference of cumulative incidence of relapse in patients with or without KIT mutation. **(f)** The difference of cumulative incidence of relapse in patients with or without FLT3 mutation. **(g)** The difference of cumulative incidence of relapse in patients with or without NRAS mutation. **(h)** The difference of cumulative incidence of relapse in patients with or without loss of X or Y.

**
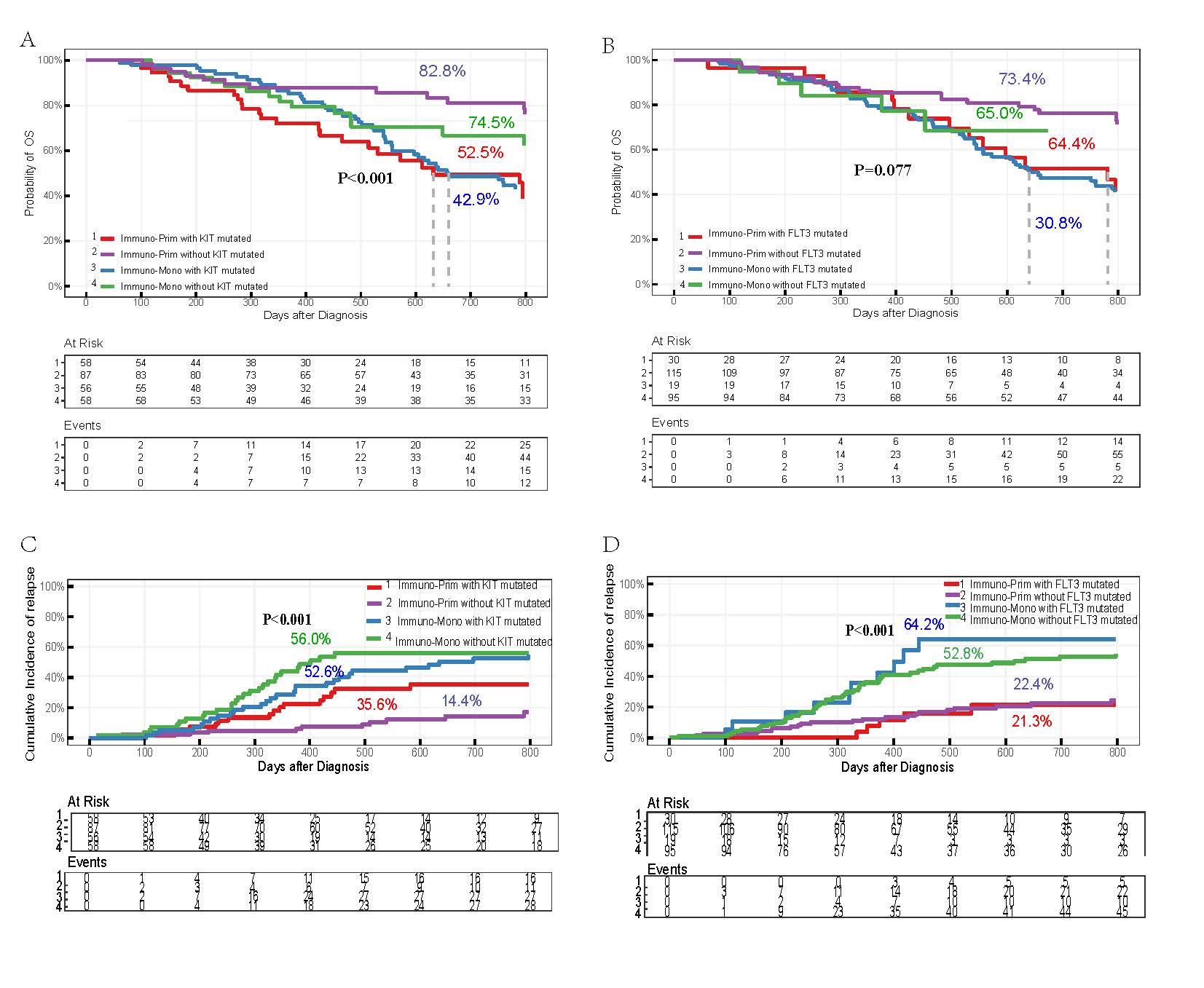
**

**Supplementary figure 2. Impact of differentiation stages combined with gene mutations on clinical outcomes in the entire cohort.**

**(a)** The difference of overall survival in patients with or without KIT mutation based on immunophenotypic stratification. **(b)** The difference of overall survival in patients with or without FLT3 mutation based on immunophenotypic stratification. **(c)** the difference of cumulative incidence of relapse in patients with or without KIT mutation based on immunophenotypic stratification. **(d)** the difference of cumulative incidence of relapse in patients with or without FLT3 mutation based on immunophenotypic stratification.


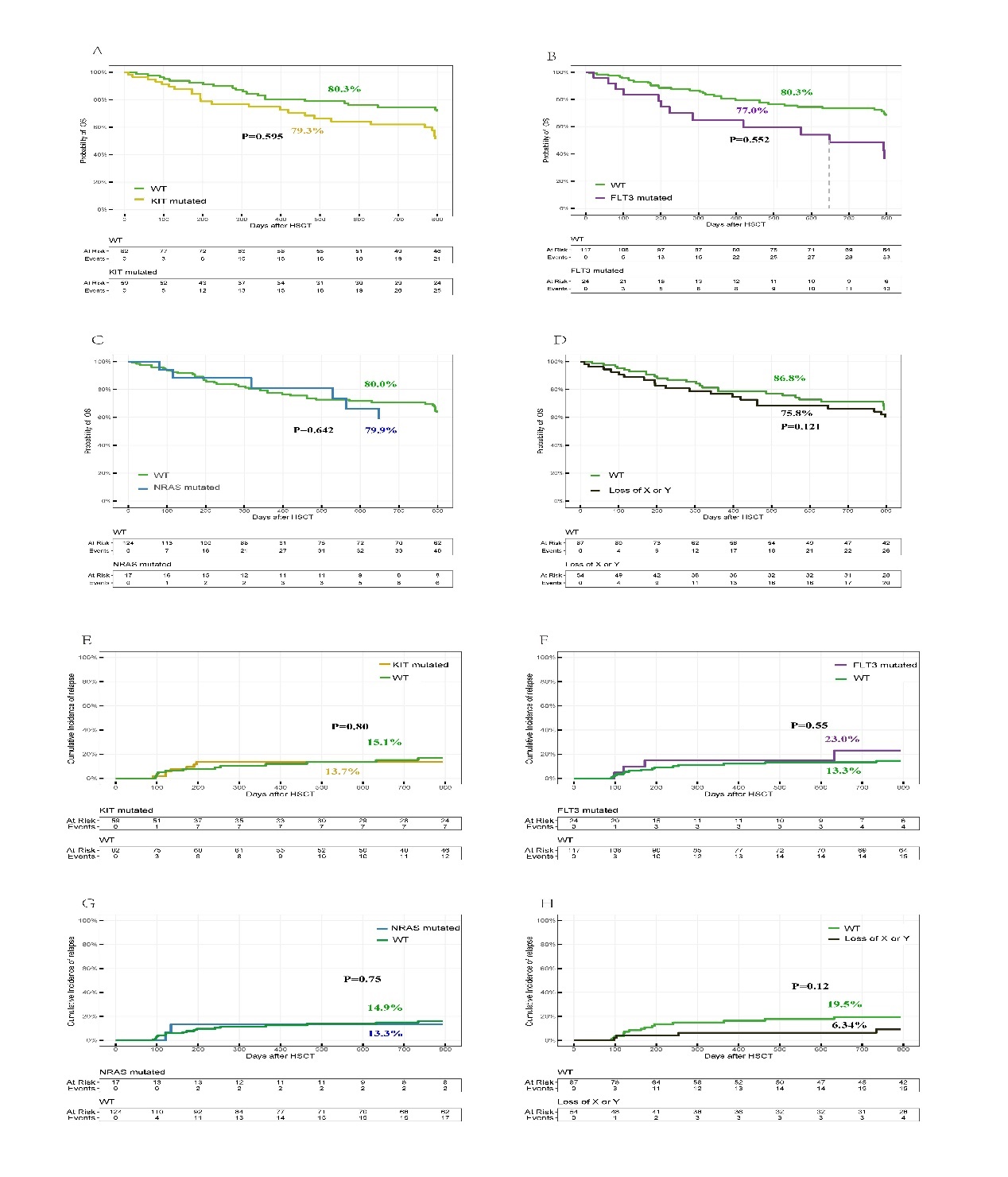


**Supplementary figure 3. Impact of gene mutations on clinical outcomes in the allo-HSCT group.**

**(a)** The difference of overall survival in patients with or without KIT mutation. **(b)** The difference of overall survival in patients with or without FLT3 mutation. **(c)** The difference of overall survival in patients with or without NRAS mutation. **(d)** The difference of overall survival in patients with or without loss of X or Y. **(e)** the difference of cumulative incidence of relapse in patients with or without KIT mutation. **(f)** the difference of cumulative incidence of relapse in patients with or without FLT3 mutation. **(g)** the difference of cumulative incidence of relapse in patients with or without NRAS mutation. **(h)** the difference of cumulative incidence of relapse in patients with or without loss of X or Y.

**
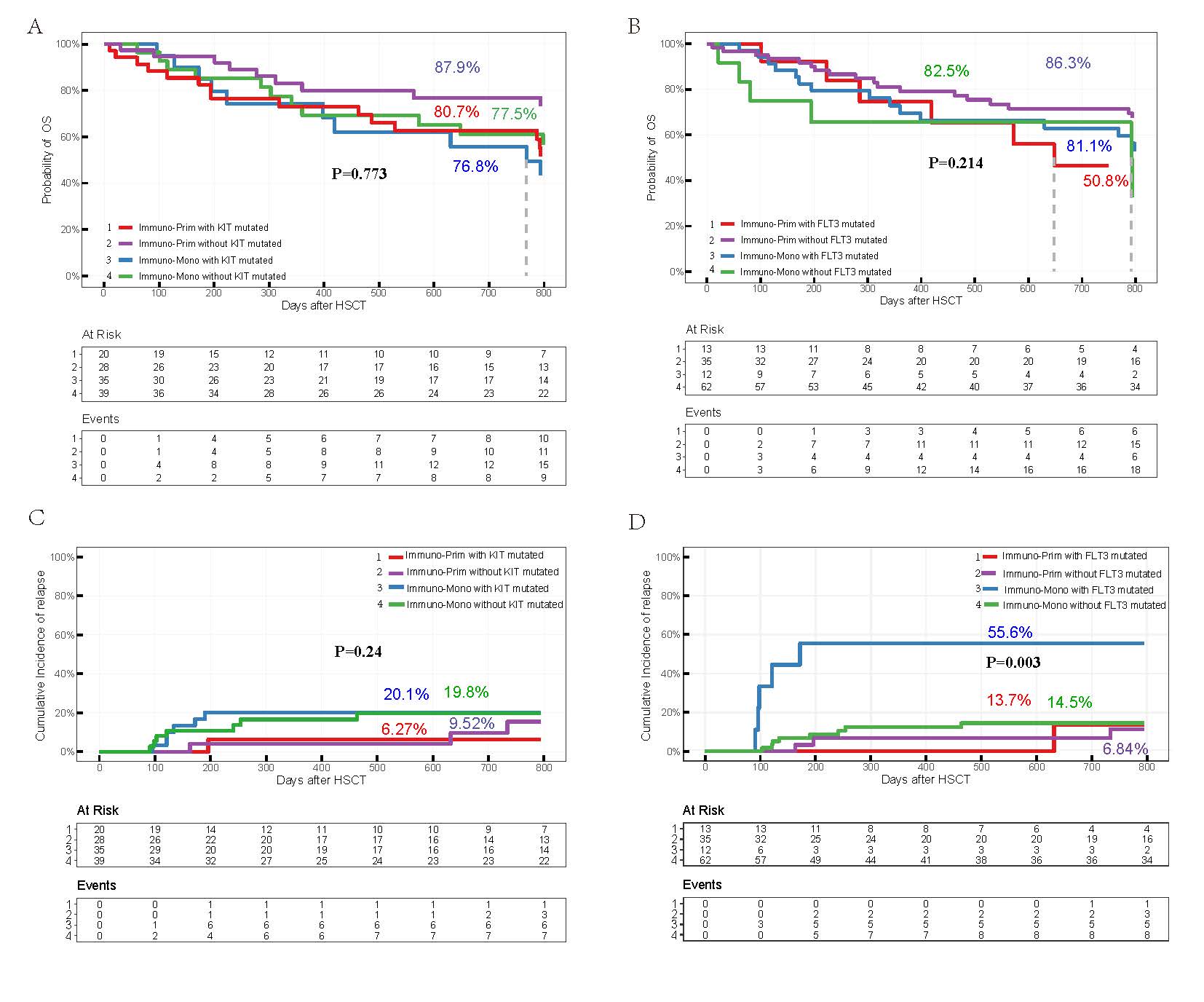
 Supplementary figure 4. Impact of** **differentiation stages combined with gene mutations on clinical outcomes in the allo-HSCT group.**

**(a)** The difference of overall survival in patients with or without KIT mutation based on immunophenotypic stratification. **(b)** The difference of overall survival in patients with or without FLT3 mutation based on immunophenotypic stratification. **(c)** The difference of cumulative incidence of relapse in patients with or without KIT mutation based on immunophenotypic stratification. **(d)** The difference of cumulative incidence of relapse in patients with or without FLT3 mutation based on immunophenotypic stratification.

**Supplementary table 1.** The panel of 92 genes for myeloid mutation assessment using next generation sequencing

| ABL1 | CDKN2A | GATA1 | KMT2D | PHF6 | SMC3 |
| --- | --- | --- | --- | --- | --- |
| AKT1 | CEBPA | GATA2 | KRAS | PIK3CA | SMO |
| AKT2 | CSF1R | GATA3 | MET | PML | SRC |
| AKT3 | CSF3R | HRAS | MPL | PRDM1 | SRSF2 |
| ANKRD26 | CUX1 | IDH1 | MSH2 | PTCH1 | STAG2 |
| APC | DDX41 | IDH2 | MTOR | PTEN | STAT3 |
| ASXL1 | DNMT3A | IKZF1 | MYC | PTPN11 | STAT5A |
| ATM | EP300 | JAK1 | MYH11 | RAD21 | STAT5B |
| ATRX | ERG | JAK2 | NF1 | RARA | STAT6 |
| BCL2 | ETV6 | JAK3 | NPM1 | RB1 | TET2 |
| BCOR | EWSR1 | KDM2B | NRAS | RUNX1 | TP53 |
| BCR | EZH2 | KDM5A | NSD1 | RUNX1T1 | U2AF1 |
| CALR | FGFR1 | KDM6A | NUP98 | SETBP1 | WT1 |
| CBFB | FIP1L1 | KIT | PDGFRA | SF3B1 | XPO1 |
| CBL | FLT3 | KMT2A | PDGFRB | SMC1A | ZRSR2 |

**Supplementary table 2.** Characteristics of AML with t (8;21) patients in Immuno-Prim and Immuno-Mono group based on immunophenotypic stratification

| Variable | Immuno-Prim Number(%)/median(range)  N=160 | Immuno-Mono Number(%)/median(range)  N=119 | P value |
| --- | --- | --- | --- |
| Male | 83 (51.88%) | 58 (48.74 %) | 0.604 |
| Female | 77 (48.13%) | 61 (51.26%) |  |
| Median age (years) | 48(15-82) | 42 (5-80) | 0.016 |
| WBC at diagnosis, 10^9^ L^–1^ | | | |
| <20 | 130 (81.25%) | 87 (73.11%) | 0.118 |
| ≥20 | 26 (16.25%) | 28 (23.53%) |  |
| FAB classification | |  |  |
| Didn’t exceed the 20% blasts without FAB classification | 7 (4.38%) | 4 (3.36%) | 0.905 |
| M0 | 1 (0.63%) | 0(0.00%) | 1.000 |
| M1 | 2 (1.25%) | 1 (0.84%) | 1.000 |
| M2 | 101 (63.13%) | 68(57.14%) | 0.312 |
| M4 | 6 (3.75%) | 11 (9.24%) | 0.058 |
| M5 | 40 (25.00%) | 35 (29.41%) | 0.411 |
| Cell source re-classification based on immunophenotypic stratification | | |  |
| Immuno-Prim | 160 (100.00%) |  |  |
| MPP | 68 (42.5%) |  |  |
| LMPP | 49 (30.63%) |  |  |
| CMP | 43 (26.88%) |  |  |
| Immuno-Mono |  | 119 (100.00 %) |  |
| GMP |  | 84 (70.59%) |  |
| GP/MP |  | 35 (29.41%) |  |
| Karyotype | |  |  |
| t(8;21)(q22;q22) | 128 (80.00%) | 96 (80.67%) | 0.889 |
| t(8;21)(q22;q22),-Y | 40 (25.00%) | 26 (21.85%) | 0.677 |
| t(8;21)(q22;q22),-X | 19 (11.88%) | 15 (12.61%) |  |
| Normal karyotype | 18 (11.25%) | 9 (7.56%) | 0.303 |
| Complex karyotype | 10 (6.25%) | 10 (8.40%) | 0.490 |
| Others | 4 (2.50%) | 4 (3.36%) | 0.727 |
| Gene mutations |  | | |
| KIT | 63 (39.38%) | 58 (48.74%) | 0.119 |
| FLT3 | 32 (20.00%) | 20 (16.81%) | 0.498 |
| NRAS | 27 (16.88%) | 12 (10.08%) | 0.106 |
| TET2 | 20 (12.50%) | 19 (15.97%) | 0.409 |
| ASXL1 | 19 (11.88%) | 18(15.13%) | 0.428 |
| Median follow up (days) | 611(?) | 1118(?) |  |

**Supplementary table 3.** The difference of gene mutations in AML patients with t (8;21) between Immuno-Prim and Immuno-Mono groups based on immunophenotypic stratification

| Variable | Immuno-Prim Number(%)/median(range)  N=160 | Immuno-Mono Number(%)/median(range)  N=119 | P value |
| --- | --- | --- | --- |
| KIT | 63(39.4%) | 58(48.7%) | 0.119 |
| FLT3 | 32(20.0%) | 20(16.8%) | 0.498 |
| CSF3R | 13(8.1%) | 7(5.9%) | 0.473 |
| CEBPA | 0(0.0%) | 1(0.8%) | 0.427 |
| RUNX1 | 0(0.0%) | 0(0.0%) | 1.000 |
| ETV6 | 3(1.9%) | 2(1.7%) | 0.637 |
| EVI1 | 0(0.0%) | 1(0.8%) | 0.427 |
| GATA2 | 4(2.5%) | 4(3.4%) | 0.727 |
| NPM1 | 0(0.0%) | 0(0.0%) | 1.000 |
| JAK1 | 9(5.6%) | 3(2.5%) | 0.206 |
| JAK2 | 11(6.9%) | 10(8.4%) | 0.632 |
| JAK3 | 4(2.5%) | 4(3.4%) | 0.727 |
| NRAS | 27(16.9%) | 12(10.1%) | 0.106 |
| KRAS | 12(7.5%) | 6(5.0%) | 0.409 |
| CBL | 10(6.3%) | 4(3.4%) | 0.274 |
| PTPN11 | 3(1.9%) | 1(0.8%) | 0.639 |
| NF1 | 1(0.6%) | 2(1.7%) | 0.577 |
| TET2 | 20(12.5%) | 19(16.0%) | 0.409 |
| IDH2 | 4(2.5%) | 3(2.5%) | 1.000 |
| IDH1 | 2(1.3%) | 3(2.5%) | 0.654 |
| DNMT3A | 2(1.3%) | 6(5.0%) | 0.076 |
| ASXL2 | 9(5.6%) | 9(7.6%) | 0.515 |
| ASXL1 | 19(11.9%) | 18(15.1%) | 0.428 |
| BCOR | 4(2.5%) | 1(0.8%) | 0.398 |
| BCORL1 | 0(0.0%) | 2(1.7%) | 0.181 |
| EZH2 | 7(4.4%) | 5(4.2%) | 0.944 |
| EP300 | 1(0.6%) | 1(0.8%) | 1.000 |
| PHF6 | 3(1.9%) | 4(3.4%) | 0.465 |
| TP53 | 3(1.9%) | 4(3.4%) | 0.465 |
| U2AF1 | 1(0.6%) | 0(0.0%) | 1.000 |
| STAG2 | 3(1.9%) | 1(0.8%) | 0.639 |
| SMC3 | 4(2.5%) | 3(2.5%) | 1.000 |
| SMC1A | 6(3.8%) | 4(3.4%) | 1.000 |
| SRSF2 | 1(0.6%) | 0(0.0%) | 1.000 |
| SF3B1 | 0(0.0%) | 2(1.7%) | 0.181 |
| ZRSR2 | 0(0.0%) | 1(0.8%) | 0.427 |
| RAD21 | 14(8.8%) | 11(9.2%) | 0.886 |
| EPPK1 | 2(1.3%) | 2(1.7%) | 1.000 |
| Titin | 2(1.3%) | 9(7.6%) | **0.018** |
| MUC16 | 3(1.9%) | 5(4.2%) | 0.292 |
| CROCC | 3(1.9%) | 1(0.8%) | 0.639 |
| ZBTB7A | 1(0.6%) | 0(0.0%) | 1.000 |

**Supplementary table 4.** Impact of differentiation stages on result of induction chemotherapy based on immunophenotypic stratification

IA, idarubicin cytarabine; DA, daunorubicin cytarabine; HAA, homoharringtonine, cytarabine and aclarubicin

| chemotherapy regimen | entire group, n | CR (n) | CR rate | P value | Immuno-Prim group, n | CR (n) | CR rate | Immuno-Mono group, n | CR (n) | CR rate | P value (Prim vs. Mono) |
| --- | --- | --- | --- | --- | --- | --- | --- | --- | --- | --- | --- |
| IA | 149 | 110 | 73.83% | 0.075 | 49 | 34 | 69.39% | 58 | 43 | 74.14% | 0.586 |
| DA | 47 | 38 | 80.85% |  | 25 | 21 | 84.00% | 20 | 16 | 80.00% | 1.000 |
| HAA | 38 | 27 | 71.05% |  | 14 | 11 | 78.57% | 20 | 13 | 65.00% | 0.637 |
| VEN based | 73 | 44 | 60.27% |  | 57 | 35 | 61.40% | 16 | 9 | 56.25% | 0.710 |
| venetoclax and azacitidine | 26 | 7 | 26.92% |  | 19 | 5 | 26.32% | 7 | 2 | 28.578% | 1.000 |

**Supplementary table 5.** Univariate analysis of clinical outcomes of AML patients with t(8;21)

| Variable | Entire group | | | | Transplant group | | | |
| --- | --- | --- | --- | --- | --- | --- | --- | --- |
|  | OS | P value | CIR | P value | OS | P value | CIR | P value |
| Age (≥44), y | 53.1%(43.5-62.7) | **<0.001** | 36.4%(27.6-45.4) | 0.61 | 70.1%(54.5-85.8) | 0.223 | 17.2%(6.8-31.4) | 0.83 |
| Age (<44), y | 80.2%(73.5-86.9) |  | 35.0%(27.2-42.9) |  | 83.5%(75.5-91.5) |  | 13.9%(7.5-22.1) |  |
| WBC (≥20), ×109/L | 58.9%(45.8-72.0) | 0.052 | 47.1%(33.4-59.6) | 0.13 | 72.7%(57.4-88.0) | 0.129 | 27.3%(13.2-43.4) | **0.032** |
| WBC (<20), ×109/L | 71.2% (64.7-77.7) |  | 32.5%(26.1-39.1) |  | 82.6%(74.6-90.6) |  | 10.1%(4.9-17.4) |  |
| Complex karyotype (Yes) | 64.8%(41.7-87.9) | 0.325 | 70.4%(34.4-89.1) | **0.016** | 88.9%(68.3-100.0) | 0.665 | 11.1%(0.5-40.6) | 0.056 |
| Complex karyotype (No) | 68.7%(62.6-74.8) |  | 33.5%(27.6-39.5) |  | 79.1%(86.7-71.5) |  | 14.9%(9.1-22.2) |  |
| Pre-HSCT MRD - | - | - | - | - | 82.0%(73.4-90.6) | 0.069 | 10.0%(4.6-17.8) | **0.006** |
| Pre-HSCT MRD + | - | - | - | - | 75.7%(62.4-89.0) |  | 23.7%(12.0-37.7) |  |
| First remission bridge to HSCT | - | - | - | - | 81.8%(73.4-90.2) | 0.100 | 9.3%(4.3-16.7) | **0.002** |
| Post relapse bridge to HSCT | - | - | - | - | 75.6%(61.7-89.5) |  | 26.8%(13.7-41.8) |  |
| FAB-M0/1/2 | 67.8%(60.5-75.1) | 0.333 | 36.2%(28.8-43.5) | 0.76 | 83.2%(74.8-91.6) | 0.230 | 11.7%(5.7-20.1) | 0.093 |
| FAB-M4/5 | 66.8%(56.2-77.4) |  | 35.3%(24.8-45.9) |  | 70.0%(54.3-85.7) |  | 25.1%(12.2-40.3) |  |
| Immuno-Prim | 71.4%(62.8-80.0) | 0.133 | 22.2%(14.9-30.4) | **<0.001** | 85.9%(74.3-97.5) | 0.367 | 6.97%(1.1-20.6) | **0.037** |
| Immuno-Mono | 59.8%(50.0-69.6) |  | 54.7%(44.1-64.1) |  | 77.2%(65.2-89.2) |  | 21.6%(11.4-33.8) |  |
| KIT (mut ) | 50.8%(40.8-60.8) | **<0.001** | 44.9%(35.2-54.2) | **0.008** | 79.3%(67.7-90.9) | 0.595 | 13.7%(5.9-24.7) | 0.80 |
| KIT (wt) | 80.2%(73.7-86.7) |  | 29.3%(22.2-36.8) |  | 80.3%(70.9-89.7) |  | 15.1%(8.0-24.4) |  |
| FLT3 (mut ) | 54.2%(37.9-70.5) | 0.098 | 35.6%(21.1-50.4) | 0.73. | 77.0%(57.0-97.0) | 0.552 | 23.0%(6.2-46.1) | 0.55 |
| FLT3 (wt) | 71.0%(64.9-77.1) |  | 35.6%(29.2-42.0) |  | 80.3%(72.5-88.1) |  | 13.3%(7.6-20.6) |  |
| NRAS (mut ) | 71.3%(55.0-87.6) | 0.587 | 24.8%(11.4-40.8) | 0.22 | 80.0%(59.8-100.0) | 0.642 | 13.3%(2.0-35.4) | 0.75 |
| NRAS (wt) | 68.2%(61.9-74.5) |  | 37.2%(30.9-43.6) |  | 79.9%(72.1-87.7) |  | 14.9%(8.9-22.4) |  |
| Loss of X or Y (Yes) | 69.2%(68.2-70.2) | 0.886 | 33.8%(24.2-43.6) | 0.34 | 75.9%(66.1-85.7) | 0.121 | 6.34%(1.6-15.8) | 0.12 |
| Loss of X or Y (No) | 67.9%(67.2-68.6) |  | 36.8%(29.4-44.2) |  | 86.8%(76.8-96.8) |  | 19.5%(11.4-29.2) |  |
| Immuno-Prim with KIT mut | 52.5%(36.8-68.2) | **<0.001** | 35.6%(21.5-50.0) | **<0.001** | 80.7% (60.9-100.0) | 0.773 | 6.27%(0.4-25.6) | 0.24 |
| Immuno-Prim with KIT wt | 82.8%(73.6-92.0) |  | 14.4%(7.1-24.1) |  | 87.9%(75.0-100.0) |  | 9.52%(1.5-27.0) |  |
| Immuno-Mono with KIT mut | 42.9%(28.0-57.8) |  | 56.0%(40.3-69.1) |  | 77.5%(61.4-93.6) |  | 20.1%(8.0-36.2) |  |
| Immuno-Mono with KIT wt | 74.5%(62.5-86.5) |  | 52.6%(37.8-65.5) |  | 76.8%(62.5-91.1) |  | 19.8%(8.5-34.4) |  |
| Immuno-Prim with FLT3mut | 64.4%(43.4-85.4) | 0.077 | 21.3%(7.3-40.1) | **<0.001** | 82.5%(60.4-100.0) | 0.214 | 13.7%(0.5-48.1) | **0.003** |
| Immuno-Prim with FLT3 wt | 73.4%(64.2-82.6) |  | 22.4%(14.3-31.7) |  | 86.3%(73.8-98.8) |  | 6.84%(1.2-20.0) |  |
| Immuno-Mono with FLT3 mut | 30.8%(6.3-55-3) |  | 64.2%(32.5-84.0) |  | 50.8%(16.1-85.5) |  | 55.6%(17.5-82.0) |  |
| Immuno-Mono with FLT3 wt | 65.0%(54.6-75.4) |  | 52.85(41.3-63.0) |  | 81.1%(70.5-91.7) |  | 14.5%(6.7-25.2) |  |
